# Financial Incentives for Smoking Cessation Among Socioeconomically Disadvantaged Adults: A Randomized Clinical Trial

**DOI:** 10.1001/jamanetworkopen.2024.18821

**Published:** 2024-07-02

**Authors:** Darla E. Kendzor, Michael S. Businelle, Summer G. Frank-Pearce, Joseph J. C. Waring, Sixia Chen, Emily T. Hébert, Michael D. Swartz, Adam C. Alexander, Munjireen S. Sifat, Laili Kharazi Boozary, David W. Wetter

**Affiliations:** 1 Department of Family and Preventive Medicine, University of Oklahoma Health Sciences Center, Oklahoma City; 2 TSET (Tobacco Settlement Endowment Trust) Health Promotion Research Center, Stephenson Cancer Center, University of Oklahoma Health Sciences Center, Oklahoma City; 3 Department of Biostatistics and Epidemiology, Hudson College of Public Health, University of Oklahoma Health Sciences Center, Oklahoma City; 4 Department of Mental Health, Bloomberg School of Public Health, The Johns Hopkins University, Baltimore, Maryland; 5 Department of Health Promotion and Behavioral Sciences, School of Public Health, University of Texas Health Science Center at Houston, Austin; 6 Department of Biostatistics and Data Science, School of Public Health, University of Texas Health Science Center at Houston, Houston; 7 Department of Medical Oncology, Sidney Kimmel Cancer Center, Thomas Jefferson University, Philadelphia, Pennsylvania; 8 Department of Population Health Sciences, Huntsman Cancer Institute, University of Utah, Salt Lake City

## Abstract

**Importance:**

Socioeconomically disadvantaged individuals (ie, those with low socioeconomic status [SES]) have difficulty quitting smoking and may benefit from incentive-based cessation interventions.

**Objectives:**

To evaluate the impact of incentivizing smoking abstinence on smoking cessation among adults with low SES.

**Design, Setting, and Participants:**

This study used a 2-group randomized clinical trial design. Data collection occurred between January 30, 2017, and February 7, 2022. Participants included adults with low SES who were willing to undergo smoking cessation treatment. Data were analyzed from April 18, 2023, to April 19, 2024.

**Interventions:**

Participants were randomized to usual care (UC) for smoking cessation (counseling plus pharmacotherapy) or UC plus abstinence-contingent financial incentives (UC plus FI).

**Main Outcomes and Measures:**

The primary outcome was biochemically verified 7-day point prevalence smoking abstinence (PPA) at 26 weeks after the quit date. Secondary outcomes included biochemically verified 7-day PPA at earlier follow-ups, 30-day PPA at 12 and 26 weeks, repeated 7-day PPA, and continuous abstinence. Multiple approaches were employed to handle missing outcomes at follow-up, including categorizing missing data as smoking (primary), complete case analysis, and multiple imputation.

**Results:**

The 320 participants had a mean (SD) age of 48.9 (11.6) and were predominantly female (202 [63.1%]); 82 (25.6%) were Black, 15 (4.7%) were Hispanic, and 200 (62.5%) were White; and 146 (45.6%) participated during the COVID-19 pandemic. Overall, 161 were randomized to UC and 159 were randomized to UC plus FI. After covariate adjustment with missing data treated as smoking, assignment to UC plus FI was associated with a greater likelihood of 7-day PPA at the 4-week (adjusted odds ratio [AOR], 3.11 [95% CI, 1.81-5.34]), 8-week (AOR, 2.93 [95% CI, 1.62-5.31]), and 12-week (AOR, 3.18 [95% CI, 1.70-5.95]) follow-ups, but not at the 26-week follow-up (22 [13.8%] vs 14 [8.7%] abstinent; AOR, 1.79 [95% CI, 0.85-3.80]). However, the association of group assignment with smoking cessation reached statistical significance at all follow-ups, including 26 weeks, with multiple imputation (37.37 [23.5%] in the UC plus FI group vs 19.48 [12.1%] in the UC group were abstinent; AOR, 2.29 [95% CI, 1.14-4.63]). Repeated-measures analyses indicated that participants in the UC plus FI group were significantly more likely to achieve PPA across assessments through 26 weeks with all missing data estimation methods. Other secondary cessation outcomes also showed comparable patterns across estimation methods. Participants earned a mean (SD) of $72 ($90) (of $250 possible) in abstinence-contingent incentives. Participation during the COVID-19 pandemic reduced the likelihood of cessation across assessments.

**Conclusions and Relevance:**

In this randomized clinical trial, incentivizing smoking cessation did not increase cessation at 26 weeks when missing data were treated as smoking; however, the UC plus FI group had greater odds of quitting at follow-ups through 12 weeks. Cessation rates were higher for the UC plus FI group at all follow-ups through 26 weeks when multiple imputation was used to estimate missing outcomes.

**Trial Registration:**

ClinicalTrials.gov Identifier: NCT02737566

## Key Points

**Question:**

Does incentivizing smoking abstinence combined with standard counseling and pharmacotherapy increase the likelihood of long-term smoking cessation among socioeconomically disadvantaged adults?

**Findings:**

This randomized clinical trial of 320 participants compared usual care (counseling and pharmacotherapy) with usual care plus modest financial incentives for biochemically verified smoking abstinence on long-term smoking cessation outcomes. Participants assigned to the incentive-based intervention were not more likely to achieve biochemically verified smoking cessation at 26-week follow-up (the primary outcome), but they were more likely to achieve cessation at all key follow-ups through 12 weeks.

**Meaning:**

Incentive-based smoking cessation treatment improved cessation rates among socioeconomically disadvantaged adults.

## Teaser

This randomized clinical trial compares the long-term effects of adjunctive, low-cost financial incentives plus usual care for smoking cessation relative to usual care counseling and pharmacotherapy alone among adults with low socioeconomic status.

## Introduction

Smoking causes nearly 20% of all cancers, 30% of all cancer deaths, and 80% of lung cancer deaths.^1,2^ Smoking is also associated with mortality from numerous other causes, including ischemic heart disease and chronic obstructive pulmonary disease.^3^ Although smoking prevalence has declined to 11.5% among US adults, at least 20% of those with Medicaid or no health insurance continue to smoke.^4^ Socioeconomically disadvantaged adults (ie, those with low socioeconomic status [SES]) are less likely to quit smoking^5,6,7,8,9,10,11^ due to contextual factors such as stress and/or adversity and smoking-conducive environments.^12,13,14,15^ The COVID-19 pandemic has had a particularly adverse impact on the health of individuals with lower SES and those who smoke.^16,17,18,19,20^

Contingency management, the tangible reinforcement of abstinence and related outcomes, is effective for promoting drug and alcohol abstinence among individuals with substance use disorders.^21,22,23^ Contingency management interventions are based on behavioral principles and operant conditioning.^24,25^ Specifically, desired outcomes (eg, abstinence) that are positively reinforced (eg, via financial incentives [FI]), are more likely to recur. A 2019 review^26^ concluded that offering incentives for smoking cessation improves long-term abstinence rates even after discontinuing incentives. Research with adults with lower SES has shown that offering small, escalating FI for smoking abstinence, when delivered as an adjunct to standard clinic-based smoking cessation treatment, dramatically increases short-term cessation.^27^

The purpose of this study was to compare the longer-term effects of adjunctive, low-cost FI for smoking cessation relative to usual care (UC) counseling and pharmacotherapy alone among adults with low SES. We hypothesized that individuals assigned to the incentives-based intervention would achieve higher rates of smoking abstinence over 26 weeks than those assigned to UC. The overlap of the study with the COVID-19 pandemic provided an opportunity to evaluate the influence of the pandemic on smoking cessation.

## Methods

This randomized clinical trial was approved by the institutional review board of the University of Oklahoma Health Sciences Center and followed the Consolidated Standards of Reporting Trials (CONSORT) reporting guideline. Written informed consent was obtained from all participants. Participants were enrolled between January 30, 2017, and August 3, 2021. Final follow-ups were completed by February 7, 2022.

### Participants

**Figure 1.**
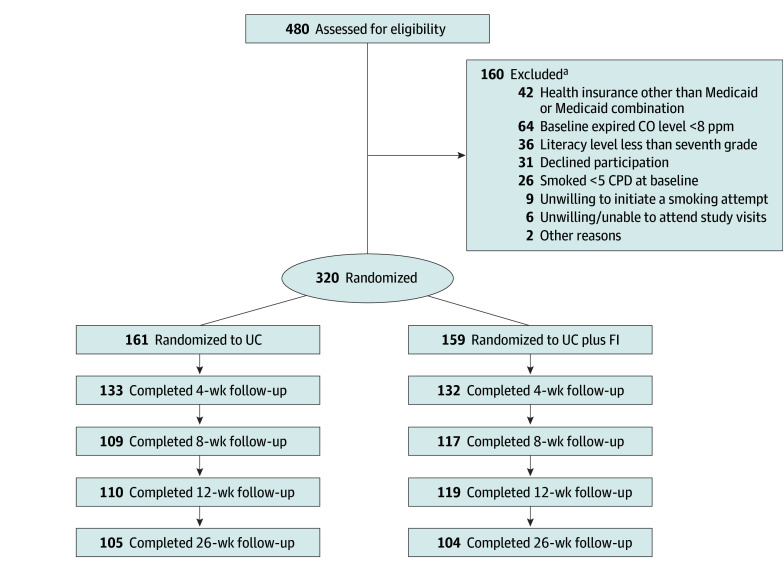
Study Flow Diagram CO indicates carbon monoxide; CPD, cigarettes per day; FI, financial incentives; and UC, usual care. ^a^Participants could have more than 1 reason for exclusion.

Individuals were eligible to participate if they (1) were uninsured or had Medicaid insurance, (2) demonstrated greater than 6th grade English literacy^28^ (necessary to complete study questionnaires), (3) were willing to initiate a smoking cessation attempt, (4) were at least 18 years of age, (5) had an expired carbon monoxide (CO) level of at least 8 ppm, (6) smoked at least 5 cigarettes per day, (7) were willing to attend study visits, and (8) were a US citizen or permanent resident (due to taxation-related university policies). Of the 480 interested individuals assessed for study eligibility, 160 were excluded (Figure 1). Excluded vs enrolled individuals were more likely to be Black or African American (51 of 132 [38.6%] excluded [28 had missing race] vs 82 of 320 [25.6%] enrolled), and there were no differences in terms of sex, ethnicity, or age. Note that a higher proportion of Black or African American individuals (11 of 51 [21.6%]) reported smoking fewer than 5 cigarettes per day compared with White individuals (4 of 69 [5.8%]) and individuals of other race (2 of 12 [16.7%]). Thus, low smoking level is a key reason why Black or African American individuals were more likely to be excluded from study participation. Participants (N = 320) were referred to the Tobacco Treatment Research Program (TTRP; a campus-based tobacco cessation clinic in Oklahoma City, Oklahoma)^29^ via electronic health record (n = 127), friend, family, or word of mouth (n = 80), clinician referral (n = 35), Trialfacts^30^ (n = 21), social media (n = 17), or other sources (n = 40). Treatment delivery and data collection for enrolled participants took place at the TTRP.

### Procedure

The study used a 2-group parallel randomized clinical trial design. Participants were randomly assigned (1:1 ratio) to UC alone (n = 161) or UC plus FI for smoking abstinence (n = 159). A study statistician (M.D.S.) generated a randomization table using the SAS, version 9.4, proc survey select package and a uniform random number generator. Blocked randomization was used with 40 blocks of 8 participants (4 UC and 4 UC plus FI per block). Study staff assigned newly enrolled participants to treatment group based on the randomization table at the baseline visit. Participants were scheduled to quit smoking 1 week after baseline and were followed up weekly through 4 weeks after the quit day via Research Electronic Data Capture (REDCap)^31,32^ assessments. Follow-up assessments were scheduled for 8, 12, and 26 weeks after the quit day. Participants were asked to provide a breath CO sample at all assessments. Participants were compensated for study assessments via department store gift cards at the time of completion. Compensation was $50 for completion of the baseline (prequit) assessment, $30 for each weekly assessment from the quit day through 4 weeks after the quit day, and $40 for follow-up assessments at 8, 12, and 26 weeks. Participants were asked to attend all study appointments in person at the TTRP until the onset of the COVID-19 pandemic. From March 16 to May 10, 2020, participants were asked to remotely complete web-based REDCap surveys, submit CO breath samples remotely via breath monitor (iCO; Bedfont), and complete counseling sessions by telephone or video call. Relatively few counseling sessions were completed remotely (144 of 1540 sessions [9.4%]), with only 57 participants (17.8%) completing at least 1 remote session (of those, 30 [52.6%] completed only 1 remote session). Study visits resumed in person May 11, 2020. However, those with a positive COVID-19 test result or symptoms and those not comfortable attending in-person visits completed study activities remotely. The study protocol is available in Supplement 1.

#### Usual Care

Participants were scheduled for a prequit counseling session with a tobacco treatment specialist and offered 5 additional weekly counseling sessions starting on the quit day. Counselors were not blinded to participant group assignment. Most participants (249 [77.8%]) were provided with combination nicotine replacement therapy (NRT; nicotine patches plus gum or lozenges) free of charge until 12 weeks after the quit day. Fewer participants used single NRTs (47 [14.7%]), no medication (21 [6.6%]), or other prescription medications or combinations (3 [0.9%]). Participants who were unwilling or unable to use NRT were evaluated by a collaborating physician and prescribed other pharmacotherapies (when appropriate) that were available free of charge at a campus pharmacy. All 320 participants were offered UC, and 161 were assigned to receive UC alone.

#### UC Plus FI

Participants assigned to UC plus FI (n = 159) were offered UC and had the opportunity to earn $250 in abstinence-contingent incentives in the form of department store gift cards (this is in addition to the aforementioned incentives earned for completing study surveys). Incentives were earned for (1) self-reported abstinence since 10 pm on the evening prior to the scheduled quit day and/or self-reported abstinence during the past 7 days at weekly visits from 1 to 4 weeks after the quit day, combined with (2) a CO breath sample consistent with abstinence (CO <10 ppm on the quit day and ≤6 ppm 1-4 weeks after the quit day). Participants earned $20 for CO-confirmed abstinence on the quit day, and this amount increased by $5 with each successive weekly abstinent visit through 4 weeks after the quit day (up to $150 total). Nonabstinent participants could earn incentives at their next visit, although the amount reset to $20. At the 8- and 12-week follow-ups, participants earned $50 per visit for self-reported past 7-day smoking abstinence with an expired CO level of no greater than 6 ppm. Incentives were delivered at the time of abstinence verification.

### Measures

#### Sociodemographic and Tobacco Use Characteristics

At baseline, participants self-reported their age, biological sex, race, ethnicity, sexual orientation, gender identity, highest level of education, annual household income, insurance status, mean number of cigarettes smoked per day, and years of smoking. The Heaviness of Smoking Index (HSI)^33^ assessed cigarette dependence (scores ≥5 indicated high dependence^34^). Participants were asked to indicate their ethnicity as either Hispanic or non-Hispanic, and they selected their race from an investigator-specified list that included the following categories: American Indian or Alaska Native, Asian, Black or African American, Native Hawaiian or Other Pacific Islander, White, multiracial, or other. Race and ethnicity are key variables in tobacco research because patterns of tobacco use and cessation vary by race and ethnicity.^4,35^

#### Treatment Adherence

The Medication Adherence Questionnaire (MAQ)^36^ is a 4-item assessment of adherence to cessation medications over the past week. Scores on the MAQ may range from 0 to 4 (4 indicates high adherence). Past week medication adherence was assessed weekly from 1 to 4 weeks after the scheduled quit date. A variable was created indicating the number of weeks with high medication adherence during the first 4 weeks after the quit day (0-1 vs 2-4 weeks). The number of counseling sessions completed was summed (range, 0-6).

#### Smoking Abstinence

Quit date abstinence was defined as self-reported abstinence since the previous evening at 10 pm and a CO level of less than 10 ppm (lenient threshold due to the recency of quitting^27,37^). Seven-day point prevalence abstinence (PPA) was defined as self-reported smoking abstinence over the past 7 days and a CO level of no greater than 6 ppm (or CO ≤6 ppm alone if missing self-report [16 instances]) based on current guidelines.^38^ The a priori primary study outcome was 7-day CO-verified PPA at 26 weeks after the quit day where participants who were lost to follow-up were considered to be smoking (MS). Additional analyses were conducted where participants lost to follow-up were excluded from analyses (complete case analysis [CCA]), and multiple imputation (MI) was also used to estimate missing outcomes. Although the MI approach was not planned a priori, more recent evidence suggests that MI may be a superior method for estimating missing smoking cessation outcomes compared with the traditional MS approach,^39,40^ and loss to follow-up in the current trial prompted its use.

Key secondary assessments of 7-day PPA occurred at 4, 8, and 12 weeks after the quit day. Other secondary outcomes included 30-day PPA at 12 and 26 weeks after the quit day, defined as self-reported abstinence over the past 30 days with an expired CO level of no greater than 6 ppm. Repeated 7-day PPA considered smoking status at every assessment point starting at 1 week after the quit day and continuing through each key follow-up (weeks 1, 2, 3, 4, 8, 12, and 26 weeks after the quit day). Participants were considered continuously abstinent at 4, 8, 12, and 26 weeks after the quit day if they reported abstinence since the quit date and had expired CO levels of no greater than 6 ppm at all study appointments beginning at 1 week after the quit date and continuing through each follow-up. Cotinine test strips were initially used to provide additional confirmation of abstinence at the final follow-up but were not used after the first 76 participants because of lack of availability during the pandemic.

A sequential MI procedure with 20 imputed values^41,42^ was used to estimate missing cessation outcomes at follow-ups. Estimating 20 imputed values ensures that the estimates achieve the desired efficiency and precision.^43^ Stepwise variable selection procedures were used to select the best set of variables in the imputation models for each follow-up. The MI procedure included 27 baseline variables reflecting treatment group assignment; participation during the COVID-19 pandemic; age; sex; race and ethnicity; sexual orientation and gender identity; educational level; insurance status; income; medication type; measures of depression, stress, and positive and negative affect; number of past smoking cessation attempts; living with someone who smokes; e-cigarette use; partner status; measures of alcohol consumption and dependence; menthol preference; prequit CO level; cigarettes smoked per day; years of smoking; and HSI. Repeated assessments of MAQ, counseling attendance, smoking status, and whether previous follow-ups occurred during or before the pandemic were also included in the models.

### Statistical Analysis

Data were analyzed from April 18, 2023, to April 19, 2024. Descriptive statistics were generated to describe the study sample. Unadjusted and adjusted logistic regression analyses were conducted to compare the influence of UC plus FI relative to UC on CO-verified 7-day PPA and continuous abstinence at all key follow-up visits (26 weeks after the quit day [primary outcome] and 4, 8, and 12 weeks after the quit day [secondary outcomes]). The influence of treatment group assignment on 30-day PPA (12 and 26 weeks after the quit day) was also examined (secondary outcomes). Generalized linear mixed-model analyses used an autoregressive covariance structure to model treatment group as a variable associated with 7-day repeated-measures PPA assessed starting at 1 week after the quit day through each key follow-up (secondary outcomes). Similar exploratory models examined the effects of sociodemographic and tobacco use characteristics, treatment adherence, and whether the assessment occurred during the COVID-19 pandemic on repeated PPA. The potential interactive effects of a follow-up occurring during the pandemic with treatment group assignment on repeated measures of cessation were also evaluated. The sample size calculation (N = 320) assumed a 30% dropout rate for both treatment groups and was based on the following assumptions: (1) 10% (UC) vs 22% (UC plus FI) 7-day PPA rates at 26 weeks after the quit day; (2) equal allocation of participants between the 2 treatments; (3) a type I error rate of .05 (2-sided tests); and (4) a minimum power of 0.8.

Covariates in the adjusted analyses included pharmacologic treatment initiated at baseline (combination NRT [nicotine patch plus gum or lozenges] compared with all others [varenicline, bupropion, single NRTs, other combinations, or no medication]), race and ethnicity (non-Hispanic White compared with all other races and ethnicities), sex, age (in years), educational level (less than high school compared with at least high school), HSI score (<5 [low or moderate dependence] compared with ≥5 [high dependence]), and whether or not the follow-up assessment occurred during the COVID-19 pandemic (prepandemic [February 29, 2020, or earlier] compared with during the pandemic [March 1, 2020, or later]). Covariates reflected variables that have been empirically linked with smoking cessation in past research. Participation before vs during COVID-19 was theorized (and confirmed) to be related to smoking cessation in the current study. All analyses were completed with SAS, version 9.4 (SAS Institute Inc), and 2-sided α = .05 indicated statistical significance.

## Results

### Participant Characteristics

Among the 320 participants, 202 (63.1%) were women and 118 (36.9%) were men (mean [SD] age, 48.9 [11.6] years). In terms of race and ethnicity, 13 participants (4.1%) were American Indian or Alaska Native, 82 (25.6%) were Black, 15 (4.7%) were Hispanic, 200 (62.5%) were White, 23 (7.2%) were multiracial, and 2 (0.6%) were Native Hawaiian or Other Pacific Islander. Table 1 provides participant and treatment characteristics. One hundred forty-six participants (45.6%) had at least 1 study visit scheduled during the COVID-19 pandemic (enrolled on or after September 1, 2019).

**Table 1.** Participant Characteristics

Characteristic	Treatment group^a^		
	All (N = 320)	UC (n = 161)	UC plus FI (n = 159)
Sociodemographic at baseline			
Age, mean (SD), y^b^	48.9 (11.6)	48.9 (12.2)	49.0 (11.1)
Sex			
Female	202 (63.1)	96 (59.6)	106 (66.7)
Male	118 (36.9)	65 (40.4)	53 (33.3)
Sexual or gender minority group^c^	40 (12.5)	23 (14.3)	17 (10.7)
Race			
American Indian or Alaska Native	13 (4.1)	4 (2.5)	9 (5.7)
Black or African American	82 (25.6)	46 (28.6)	36 (22.6)
White	200 (62.5)	99 (61.5)	101 (63.5)
Multiracial or other^d^	25 (7.8)	12 (7.5)	13 (8.2)
Hispanic ethnicity	15 (4.7)	8 (5.0)	7 (4.4)
Member of racial or ethnic minority group	126 (39.4)	67 (41.6)	59 (37.1)
Educational level less than high school^b^	62 (19.4)	29 (18.0)	33 (20.8)
Annual household income <$11 000^e^	175 (54.7)	86 (53.4)	89 (56.0)
Health insurance, Medicaid or Medicaid combination^b^^,^^f^	175 (54.9)	89 (55.3)	86 (54.1)
Any study participation during COVID-19 pandemic^g^	146 (45.6)	75 (46.6)	71 (44.7)
Smoking at baseline			
Cigarettes smoked per day, mean (SD)^b^	19.1 (10.1)	19.2 (10.1)	19.1 (10.2)
Duration of smoking, mean (SD), y^b^	29.2 (13.2)	28.8 (13.7)	29.5 (12.6)
Expired CO, mean (SD), ppm	22.5 (11.6)	21.9 (11.2)	23.1 (12.1)
Heaviness of Smoking Index score ≥5 (high dependence)^b^	63 (19.7)	35 (21.7)	28 (17.6)
E-cigarette use in past 30 d	95 (29.7)	53 (32.9)	42 (26.4)
Menthol or both menthol and nonmenthol use	134 (41.9)	75 (46.6)	59 (37.1)
Treatment			
Combination NRT	249 (77.8)	127 (78.9)	122 (76.7)
Counseling sessions completed, median (IQR)^h^	6 (4.5-6)	5 (4-6)	6 (5-6)
All counseling sessions completed	170 (53.1)	80 (49.7)	90 (56.6)
High medication adherence^i^	155 (51.8)	73 (48.3)	82 (55.4)

Abbreviations: CO, carbon monoxide; FI, financial incentive; NRT, nicotine replacement therapy; UC, usual care.

^a^ Unless otherwise indicated, data are expressed as No. (%) of patients. Percentages have been rounded and may not total 100.

^b^ One participant did not provide this information.

^c^ Participants were considered sexual/gender minoritized if they identified as lesbian or gay (n = 12 [1 also identified as transgender]), bisexual (n = 19), transgender (n = 3 [2 also identified as straight/heterosexual; 1 also identified as lesbian or gay]), did not know or were not sure about their sexual orientation (n = 2 [1 was not sure whether they were transgender]), or chose not to respond (n = 5 [2 chose not to respond about whether they were transgender]). Those who identified as straight and did not identify as transgender were considered heterosexual or cisgender.

^d^ Participants of other race self-identified as Native Hawaiian or Other Pacific Islander (n = 2) or multiracial (n = 23), with the following combinations: 6 American Indian or Alaska Native and White (1 Hispanic); 4 American Indian or Alaska Native, Black, and White (1 Hispanic); 3 Black and White; 3 American Indian or Alaska Native and Black; 1 American Indian or Alaska Native, Native Hawaiian or Other Pacific Islander, and White (1 Hispanic); 1 Asian and White; and 5 multiracial but only identified their race as White (3 Hispanic). Fourteen of 23 participants (60.9%) who identified as multiracial selected American Indian or Alaska Native as one of their races.

^e^ Nine participants did not provide this information.

^f^ Participants who did not have Medicaid insurance were uninsured.

^g^ Participants enrolled between September 1, 2019, and August 3, 2021, had at least 1 study visit scheduled during the COVID-19 pandemic.

^h^ The median number of counseling sessions completed differed significantly by treatment group assignment (*P* < .05).

^i^ High medication adherence was defined as having 2 or more weeks (during the first 4 weeks after the scheduled quit date) with a score of 4 on the Medication Adherence Questionnaire, assessed only in those who were provided with medication at baseline (n = 299).

### Effect of Incentive-Based Treatment on Smoking Cessation

Complete smoking status data (self-reported smoking or self-reported and CO-verified abstinence) were available for 265 participants (82.8%) at 4 weeks of follow-up, 226 (70.6%) at 8 weeks, 229 (71.6%) at 12 weeks, and 209 (65.3%) at 26 weeks. Follow-up rates did not differ significantly by treatment group at any follow-up. However, follow-up rates differed significantly at all follow-ups based on whether the follow-up was scheduled before or during the pandemic. At 4 weeks, follow-up rates were 185 of 214 (86.4%) before COVID-19 vs 80 of 106 (75.5%) during COVID-19; at 8 weeks, 153 of 201 (76.1%) vs 73 of 119 (61.3%), respectively; at 12 weeks, 151 of 196 (77.0%) vs 78 of 124 (62.9%), respectively; and at 26 weeks, 126 of 174 (72.4%) vs 83 of 146 (56.8%), respectively. Participants who did not complete the final follow-up were younger, reported fewer years of smoking, were less likely to be female, and were more likely to have Medicaid insurance and to have participated in the study during the pandemic than those who completed the study (eTable in Supplement 2).

Cessation rates are presented by treatment group assignment in Table 2. Adjusted logistic regression analyses indicated that assignment to UC plus FI was associated with significantly greater odds of CO-verified 7-day PPA relative to UC at the 4-week (adjusted odds ratio [AOR], 3.11 [95% CI, 1.81-5.34]), 8-week (AOR, 2.93 [95% CI, 1.62-5.31]), and 12-week (AOR, 3.18 [95% CI, 1.70-5.95]) follow-ups, but not the final 26-week follow-up, when the MS approach was used (22 [13.8%] vs 14 [8.7%] abstinent; AOR, 1.79 [95% CI, 0.85-3.80]) (Table 2 and Table 3). Findings were similar when missing cessation outcomes were estimated using CCA and MI; however, the associations between treatment group assignment and smoking cessation reached statistical significance at all follow-ups, including the final 26-week follow-up when MI was used (37.37 [23.5%] in the UC plus FI group vs 19.48 [12.1%] in the UC group abstinent; AOR, 2.29 [95% CI, 1.14-4.63]) (Table 2 and Table 3). In the repeated-measures analysis of 7-day PPA, assignment to UC plus FI was associated with a greater likelihood of PPA across the entire 26-week follow-up period (weeks 1-4, 8, 12, and 26) when the MS, CCA, and MI approaches were used. The likelihood of achieving 30-day PPA was significantly greater at the 12-week follow-up among those assigned to UC plus FI relative to UC when MS, CCA, and MI were used. In addition, 30-day PPA was significantly greater in UC plus FI relative to UC at 26-week follow-up in the adjusted and unadjusted analyses when MI was used. Finally, rates of continuous abstinence were significantly greater for UC plus FI relative to UC at the 4, 8, and 12-week follow-ups with or without MI, and these differences were maintained at the 26-week follow-up with MI (Table 3).

**Table 2.** Smoking Abstinence Rates by Treatment Group Assignment at Follow-Up

Measurement	Treatment group, No. (%)											
	4-wk Follow-up			8-wk Follow-up			12-wk Follow-up			26-wk Follow-up		
	All	UC	UC plus FI	All	UC	UC plus FI	All	UC	UC plus FI	All	UC	UC plus FI
7-d PPA												
MS^a^	88 (27.5)	27 (16.8)	61 (38.4)	70 (21.9)	22 (13.7)	48 (30.2)	63 (19.7)	18 (11.2)	45 (28.3)	36 (11.3)	14 (8.7)	22 (13.8)
CCA^b^	88 (33.2)	27 (20.3)	61 (46.2)	70 (31.0)	22 (20.2)	48 (41.0)	63 (27.5)	18 (16.4)	45 (37.8)	36 (17.2)	14 (13.3)	22 (21.2)
MI^c^	103.26 (32.3)	31.07 (19.3)	72.19 (45.4)	95.63 (29.9)	30.91 (19.2)	64.71 (40.7)	80.91 (25.3)	23.99 (14.9)	56.92 (35.8)	56.85 (17.8)	19.48 (12.1)	37.37 (23.5)
30-d PPA												
MS^a^	NA	NA	NA	NA	NA	NA	58 (18.1)	17 (10.6)	41 (25.8)	34 (10.6)	13 (8.1)	21 (13.2)
CCA^b^	NA	NA	NA	NA	NA	NA	58 (25.3)	17 (15.5)	41 (34.5)	34 (16.3)	13 (12.4)	21 (20.2)
MI^c^	NA	NA	NA	NA	NA	NA	72.62 (22.7)	22.38 (13.9)	50.24 (31.6)	52.69 (16.5)	17.71 (11.0)	34.98 (22.0)
Continuous abstinence^d^	41 (12.8)	12 (7.5)	29 (18.2)	34 (10.6)	11 (6.8)	23 (14.5)	28 (8.8)	7 (4.4)	21 (13.2)	15 (4.7)	4 (2.5)	11 (6.9)
Continuous abstinence by MI^e^	42.14 (13.2)	12.88 (8.0)	29.26 (18.4)	38.31 (12.0)	12.24 (7.6)	26.08 (16.4)	29.68 (9.3)	8.86 (5.5)	20.83 (13.1)	16.74 (5.2)	3.70 (2.3)	13.04 (8.2)

Abbreviations: CCA, complete case analysis; FI, financial incentives; MI, multiple imputation; MS, participants with missing smoking cessation; NA, not applicable; PPA, carbon monoxide (CO)–verified point prevalence abstinence; UC, usual care.

^a^ Participants with missing smoking cessation follow-up outcomes were considered to be smoking (all 320 randomized participants included, 161 in the UC group and 159 in the UC plus FI group).

^b^ Includes 265 complete cases at 4 weeks, 226 at 8 weeks, 229 at 12 weeks, and 209 at 26 weeks; participants with missing smoking cessation outcomes were excluded from the analysis.

^c^ Used to estimate missing smoking cessation follow-up outcomes (all 320 randomized participants included, 161 in the UC group and 159 in the UC plus FI group).

^d^ Indicates self-reported abstinence since the quit date, CO verified at all available times (1 missing allowed) through the specified follow-up time among all randomized participants (N = 320).

^e^ Indicates self-reported and CO-verified abstinence since the quit date through the specified follow-up time among all randomized participants (N = 320). Multiple imputation was used to estimate missing smoking cessation outcomes at follow-ups.

**Table 3.** Associations Between Treatment Group Assignment and Smoking Cessation at Follow-Up

Measurement	Time after quit date							
	4 wk		8 wk		12 wk		26 wk	
	OR (95% CI)	AOR (95% CI)^a^	OR (95% CI)	AOR (95% CI)^a^	OR (95% CI)	AOR (95% CI)^a^	OR (95% CI)	AOR (95% CI)^a^
7-d PPA								
MS^b^	3.09 (1.83-5.21)^c^	3.11 (1.81-5.34)^c^	2.73 (1.56-4.80)^c^	2.93 (1.62-5.31)^c^	3.14 (1.72-5.71)^c^	3.18 (1.70-5.95)^c^	1.69 (0.83-3.43)	1.79 (0.85-3.80)
CCA^d^	3.37 (1.96-5.81)^c^	3.52 (2.00-6.21)^c^	2.75 (1.52-4.99)^c^	3.11 (1.65-5.87)^c^	3.11 (1.66-5.82)^c^	3.23 (1.67-6.26)^c^	1.74 (0.84-3.63)	1.93 (0.89-4.18)
MI^e^	3.48 (2.03-5.97)^c^	3.58 (2.03-6.30)^c^	2.89 (1.67-5.01)^c^	2.91 (1.63-5.18)^c^	3.18 (1.77-5.73)^c^	3.10 (1.69-5.67)^c^	2.24 (1.12-4.46)^c^	2.29 (1.14-4.63)^c^
Repeated 7-d PPA								
MS^b^	2.81 (1.81-4.37)^c^	2.85 (1.82-4.46)^c^	2.74 (1.80-4.17)^c^	2.79 (1.83-4.27)^c^	2.83 (1.89-4.24)^c^	2.88 (1.91-4.35)^c^	2.69 (1.82-3.98)^c^	2.72 (1.84-4.02)^c^
CCA^d^	2.95 (1.87-4.65)^c^	2.96 (1.86-4.71)^c^	2.81 (1.82-4.31)^c^	2.88 (1.85-4.48)^c^	2.85 (1.89-4.32)^c^	2.90 (1.89-4.44)^c^	2.76 (1.84-4.11)^c^	2.80 (1.87-4.21)^c^
MI^e^	2.91 (1.91-4.45)^c^	2.98 (1.94-4.59)^c^	2.81 (1.88-4.18)^c^	2.85 (1.90-4.28)^c^	2.87 (1.98-4.18)^c^	2.89 (1.98-4.23)^c^	2.75 (1.91-3.95)^c^	2.75 (1.91-3.97)^c^
30-d PPA								
MS^b^	NA	NA	NA	NA	2.94 (1.59-5.45)^c^	2.95 (1.55-5.60)^c^	1.73 (0.83-3.59)	1.86 (0.86-4.00)
CCA^d^	NA	NA	NA	NA	2.88 (1.52-5.46)^c^	2.97 (1.51-5.82)^c^	1.79 (0.84-3.80)	2.02 (0.91-4.46)
MI^e^	NA	NA	NA	NA	2.86 (1.57-5.20)^c^	2.78 (1.51-5.13)^c^	2.29 (1.09-4.80)^c^	2.32 (1.09-4.95)^c^
Continuous abstinence^f^	2.77 (1.36-5.65)^c^	2.98 (1.42-6.25)^c^	2.31 (1.08-4.91)^c^	2.51 (1.13-5.59)^c^	3.35 (1.38-8.12)^c^	3.74 (1.47-9.47)^c^	2.92 (0.91-9.36)	2.97 (0.88-10.02)
Continuous abstinence by MI^g^	2.62 (1.27-5.41)^c^	2.77 (1.30-5.88)^c^	2.41 (1.12-5.16)^c^	2.59 (1.15-5.84)^c^	2.59 (1.06-6.31)^c^	2.71 (1.15-6.95)^c^	3.86 (1.11-13.40)^c^	4.00 (1.10-14.53)^c^

Abbreviations: AOR, adjusted odds ratio (OR); CCA, complete case analysis; NA, not applicable; MI, multiple imputation; MS, missing smoking cessation considered to be smoking; PPA, carbon monoxide (CO)–verified point prevalence abstinence.

^a^ Adjusted for age (years), sex, race and ethnicity (non-Hispanic White vs all others), educational level (at least high school vs less than high school completion), Heaviness of Smoking Index score (≥5 vs <5), and use of combination nicotine replacement therapy vs other medications or no medication (assessed at baseline), and whether the study visit occurred during the COVID-19 pandemic. For the repeated measures analysis, treatment week was also included in both unadjusted and adjusted analyses.

^b^ Participants with missing smoking cessation follow-up outcomes were considered to be smoking (all 320 randomized participants included, 161 in the usual care [UC] group and 159 in the UC plus financial incentive [FI] group).

^c^ *P* < .05.

^d^ Includes 265 complete cases at 4 weeks, 226 at 8 weeks, 229 at 12 weeks, and 209 at 26 weeks. Participants with missing smoking cessation outcomes were excluded from the analysis; in the case of repeated-measures analyses, all available data were used.

^e^ Used to estimate missing smoking cessation follow-up outcomes (all 320 randomized participants included, 161 in the UC group and 159 in the UC plus FI group). Missing covariate data for age (n = 1), education (n = 1), and Heaviness of Smoking Index (n = 1) were estimated via MI; participants with missing covariate values (n = 3) were excluded from MS and CCA analyses.

^f^ Indicates self-reported abstinence since the quit date, CO verified at all available times (1 missing allowed) through the specified follow-up time among all randomized participants (N = 320).

^g^ Indicates self-reported and CO-verified abstinence since the quit date through the specified follow-up time among all randomized participants (N = 320). Multiple imputation was used to estimate missing smoking cessation outcomes at follow-ups.

### Incentives Earned

Participants assigned to UC plus FI earned a mean (SD) of $72 ( $90) (median, $20 [IQR, $0-$145]) of $250 in available abstinence-contingent incentives over the first 12 weeks after the quit day. The total amount of abstinence-contingent incentives earned was $11 465, with a mean cost per quit of $521.14 (22 quits) with MS and $309.86 (37 quits) with MI based on 7-day PPA in the UC plus FI group at the 26-week follow-up.

### Individual Characteristics Associated With Smoking Cessation

The following variables were examined in repeated measures analyses as factors associated with CO-verified 7-day PPA across assessment weeks (weeks 1-4, 8, 12, and 26) where MI was used to estimate missing smoking cessation outcomes: race and ethnicity, sexual orientation and gender identity, educational level, annual household income, insurance status, mean number of cigarettes smoked per day before the quit date, years of smoking, CO level before the quit date, HSI, and medication type. Analyses indicated that those with a household income of less than $11 000 were less likely to achieve 7-day PPA than those earning at least $11 000 across assessments (OR, 0.56 [95% CI, 0.39-0.81]). Higher baseline CO level was associated with a lower likelihood of cessation across assessments (OR, 0.98 [95% CI, 0.96-0.99]).

### COVID-19 Pandemic

**Figure 2.**
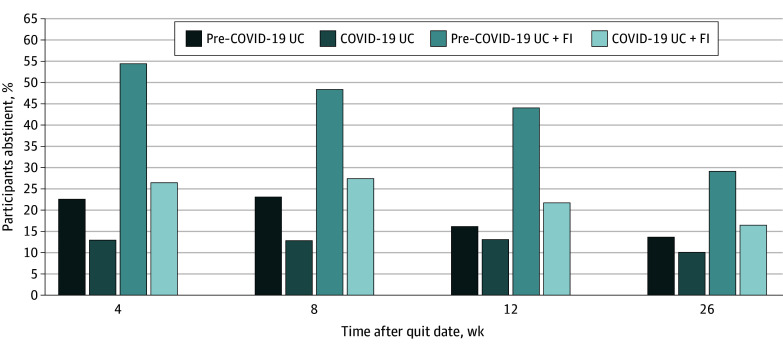
Point Prevalence Smoking Abstinence Rates by Treatment Group Assignment and Participation During the COVID-19 Pandemic Follow-ups scheduled on March 1, 2020, or later were considered to have occurred during the COVID-19 pandemic. Missing smoking cessation outcomes were estimated using multiple imputation methods. Participant sample sizes at follow-up in the usual care (UC) group before the pandemic (February 29, 2020, or earlier) were 106 at 4 weeks, 100 at 8 weeks, 96 at 12 weeks, and 86 at 26 weeks. Sample sizes for participants in the UC group during the pandemic were 55 at 4 weeks, 61 at 8 weeks, 65 at 12 weeks, and 75 at 26 weeks. Sample sizes for participants in the UC plus financial incentives (FI) group before the pandemic were 108 at 4 weeks, 101 at 8 weeks, 100 at 12 weeks, and 88 at 26 weeks. Sample sizes for participants in the UC plus FI group during the pandemic were 51 at 4 weeks, 58 at 8 weeks, 59 at 12 weeks, and 71 at 26 weeks.

Using MI to estimate missing smoking cessation outcomes, adjusted repeated-measures analyses indicated that having any assessment scheduled during the pandemic was associated with a lower likelihood of 7-day PPA across assessment weeks (weeks 1-4, 8, 12, and 26; AOR, 0.40 [95% CI, 0.28-0.58]). Treatment group assignment did not interact with whether the follow-up occurred during (vs before) the pandemic to influence smoking cessation across assessment weeks. Participants assigned to UC plus FI who completed their follow-up prior to the COVID-19 pandemic achieved the highest cessation rates (Figure 2). Study analyses were not powered to detect this interaction.

## Discussion

This study evaluated whether abstinence-contingent FI combined with UC would increase longer-term smoking cessation rates relative to UC alone among adults with lower SES. Findings indicated that the likelihood of achieving abstinence was greater among those who received abstinence-contingent incentives through the 12-week follow-up across all measures of abstinence and approaches to missing data estimation. At the 26-week follow-up, rates of 7-day PPA, 30-day PPA, and continuous abstinence were not significantly greater for those assigned to receive incentive-based treatment when MS was used, but they were significant when MI was used. Repeated PPA was significantly greater for UC plus FI through 26-week follow-up across all approaches to missing data estimation. Notably, those who had follow-ups scheduled after the onset of the COVID-19 pandemic were less likely to complete follow-up visits and achieve abstinence than those who participated before the pandemic. Overall, our findings support the use of incentive-based smoking cessation treatment to increase abstinence rates among socioeconomically disadvantaged adults.

The present findings add to the substantial body of research demonstrating that incentive-based interventions are effective for promoting smoking cessation.^26^ Primarily pilot studies have focused on socioeconomically disadvantaged populations, including patients from safety-net hospitals,^27,44^ people experiencing homelessness,^45,46,47,48,49^ adults with low income,^50^ and economically disadvantaged pregnant women.^27,45,46,47,49,50,51^ In a full-scale randomized trial with patients from a safety net hospital, Lasser et al^44^ reported that enhanced UC (ie, smoking cessation brochure, list of cessation resources), patient navigation (eg, connection with resources, brief counseling, facilitation of medication access), and incentives for biochemically confirmed abstinence at 6 ($250) and 12 months (≤$500) after enrollment increased long-term cessation rates relative to enhanced UC alone. Their study used less intensive treatment and provided relatively large incentives for longer-term abstinence, which may have practical benefits at the system level (eg, greater reach, simplicity, fewer required treatment resources).

The present study used an intervention approach that combined weekly, small-value abstinence-contingent incentives early in treatment with intensive guideline-based^52^ tobacco cessation treatment, including counseling and pharmacotherapy. With this approach, abstinence was reinforced frequently during the initial phase of cessation, when lapse is most likely. In addition, small-value incentives were relatively low cost and potentially cost-effective (ie, $72 per participant), though administrative costs were not included. Plausibly, small-value incentives may be more motivating among individuals with fewer resources compared with their counterparts with higher SES. Incentivized participants may have been more engaged in treatment because of their desire to earn incentives for quitting smoking (eg, UC plus FI participants completed more counseling sessions than UC participants) (Table 1). Repeated reinforcement for a desired outcome can shape and promote positive behavior change and may lead to increased self-efficacy and confidence about one’s ability to quit.^53^

Importantly, some research has focused on the development and evaluation of remote and mobile approaches to incentive-based smoking cessation treatment.^48,50,54,55,56^ Remotely delivered interventions may increase the reach of incentive-based interventions, which is particularly important for socioeconomically disadvantaged individuals who may experience transportation and other barriers to accessing clinic-based interventions. In addition, policy-related barriers to implementation^57^ must be addressed to increase adoption (eg, via Medicaid coverage^58^).

### Strengths and Limitations

The present study has strengths and limitations. We used a randomized clinical trial design, followed up with participants over 26 weeks, and focused on adults with low SES who experience tobacco-related health disparities. The sample also included many individuals from other groups that experience tobacco-related disparities and/or are underrepresented in research. Nevertheless, study recruitment and data collection took place in a single state, and thus, participants may be less representative of people from other areas. As is common in smoking cessation studies targeting vulnerable populations (and with the unexpected difficulties associated with the pandemic), missing outcome data posed challenges to statistical analyses. However, the traditional approaches of categorizing participants with missing follow-up data as smoking (MS), excluding them from analyses (CCA), and the more recently accepted MI approach^39,40^ were used to estimate missing cessation outcomes and illustrate how findings differed depending on assumptions about missingness. Findings were largely consistent across methods, but the effect of treatment group assignment reached significance at final follow-up primarily in analyses that used MI.

## Conclusions

In this randomized clinical trial of an incentive-based smoking cessation intervention, incorporating low-cost abstinence-contingent incentives as part of a standard smoking cessation treatment approach did not increase smoking cessation at 26 weeks relative to UC alone among socioeconomically disadvantaged adults when missing data were treated as smoking. However, secondary analyses indicated that UC plus FI was associated with long-term abstinence when MI was used to estimate missing outcomes. Future research is needed to optimize treatment component combinations and durations, characterize treatment mechanisms, and address implementation barriers. Efforts are needed to understand the mechanisms underlying the adverse effects of the pandemic on cessation and address pandemic-related cessation disparities that may have developed.

## Notes

Supplement 1.
Trial Protocol

Supplement 2. **eTable.** Comparison of the Baseline Characteristics of Participants Who Completed the Final 26-Week Follow-Up vs Those Who Did Not

Supplement 3.
Data Sharing Statement
