# Supplementary material for: Financial Incentives for Smoking Cessation Among Socioeconomically Disadvantaged Adults: A Randomized Clinical Trial
Source: JAMA Netw Open. 2024 Jul 2;7(7):e2418821. doi: 10.1001/jamanetworkopen.2024.18821 (PMC11220567; doi:10.1001/jamanetworkopen.2024.18821)
Supplement: Supplement 1. — Trial Protocol [file jamanetwopen-e2418821-s001.pdf]

Small Financial Incentives to Promote Smoking Cessation in Safety Net Hospital Patients

Principal Investigator: **Darla E. Kendzor, Ph.D.,** Stephenson Cancer Center, Department of Family and Preventive Medicine, OUHSC

Co-Investigators: **Michael Businelle, Ph.D.,** Department of Family and Preventive Medicine Stephenson Cancer Center, OUHSC

Michael Swartz, Ph.D., Department of Biostatistics, School of Public Health, The University of Texas Health Science Center, Houston, TX

David Wetter, Ph.D., Department of Psychology, Rice University, Houston, TX

Abstract
Although the prevalence of smoking has declined to 17.8% among U.S. adults, 29.2% of those living in poverty report current smoking. In addition, socioeconomic disadvantage is associated with a reduced likelihood of smoking cessation. There is mounting evidence that offering incentives for abstinence (i.e., Contingency Management [CM]) may be an effective approach for promoting short-term smoking cessation, though few studies have demonstrated longer-term effectiveness and little attention has been paid to socioeconomically disadvantaged smokers. The aims of this study are to: 1) evaluate the longer-term impact of an adjunctive, low-cost CM intervention (relative to usual care) on smoking abstinence rates among socioeconomically disadvantaged individuals participating in a clinic-based smoking cessation program, and 2) identify treatment mechanisms and contextual factors associated with cessation outcomes using both traditional and ecological momentary assessment approaches. Socioeconomically disadvantaged individuals participating in a clinic-based smoking cessation program (*N* = 320) will be randomly assigned to 1) standard care (SC; *n*=160) *or* 2) SC plus financial incentives for biochemically-confirmed abstinence (CM; *n*=160). Those randomized to the CM intervention will earn small gift cards for biochemically-verified abstinence through 12 weeks post-quit. Biochemically-verified 7-day point prevalence abstinence at 26 weeks post-quit (longer-term abstinence) will be the primary outcome variable. We also hope to gain a better understanding of CM treatment mechanisms and to identify other factors that influence cessation. Smartphones offer the benefit of “real-time” assessment, and will also be used to deliver gain-framed messages to support the CM intervention.

A. Specific Aims

Our preliminary work has indicated that offering small escalating financial incentives for smoking abstinence dramatically increases **short-term** cessation rates among socioeconomically disadvantaged smokers when incentives are included as an adjunct to the standard clinic-based smoking cessation treatment.[1,2] Additional work is needed to evaluate the influence of this CM approach in the **longer-term** with a rigorous and adequately powered trial. Thus, our long-term goal is to reduce smoking rates among socioeconomically disadvantaged individuals and to thereby reduce tobacco-related health disparities. The next step in our pursuit of this goal is to examine the effectiveness of our low-cost financial incentives paradigm on longer-term cessation at 6 months post-quit in a randomized controlled trial (*N* = 320). Specifically, 320 socioeconomically disadvantaged individuals will be randomly assigned to standard care (SC) in a clinic-based smoking cessation program *or* SC plus a 12-week financial incentives intervention (CM) for biochemically-confirmed abstinence. We also hope to gain a better understanding of CM treatment mechanisms and to identify other factors that directly influence cessation using traditional questionnaire and smartphone-based ecological momentary assessment approaches. If effective, our simple, low-cost financial incentives paradigm may be incorporated into existing tobacco cessation programs to increase cessation rates in economically disadvantaged smokers. Thus, we will pursue the following specific aims:

Aim 1. To evaluate the longer-term impact of an adjunctive, low-cost CM intervention (relative to standard care) on smoking abstinence rates among socioeconomically disadvantaged individuals participating in a clinic-based smoking cessation program. *We hypothesize that individuals randomly assigned to the adjunctive*

IRB NUMBER: 6260
IRB APPROVAL DATE: 04/08/2021
IRB NUMBER: 6260
IRB APPROVAL DATE: 04/08/2021

CM intervention will have significantly higher rates of biochemically-verified abstinence at the 26-week post-quit follow-up than those assigned to Standard Care.

Aim 2. To identify treatment mechanisms and contextual factors associated with cessation outcomes among intervention participants using both traditional and ecological momentary assessment approaches. *Potential treatment mechanisms are hypothesized to include motivation, self-efficacy, and treatment adherence. In addition, we hypothesize that several factors related to socioeconomic disadvantage will be directly associated with non-abstinence, especially greater stress/adversity, limited psychosocial resources, greater negative affect, greater nicotine dependence, and poorer treatment adherence.*

B. Background and Significance

Smoking rates are disproportionately high among socioeconomically disadvantaged individuals,[3] despite comparable numbers of quit attempts relative to smokers of higher socioeconomic status.[4] Exposure to stressors/adversity, limited psychosocial resources, greater nicotine dependence, greater negative affect, and poor adherence to smoking cessation treatments have been hypothesized to contribute to dismal smoking cessation outcomes and poor general health in socioeconomically disadvantaged populations.[5-7] Notably, contingency management (CM), or the tangible reinforcement of abstinence and other related outcomes, is one approach that has been effective for the promotion of abstinence among individuals participating in treatment for substance abuse/dependence.[8-10] There is mounting evidence that the CM approach may also be effective at promoting **short-term** smoking cessation among substance-dependent smokers, pregnant/post-partum women, and in a variety of other specific populations.[11-26] In addition, the findings of a recent meta-analysis have indicated that financial incentives interventions increase **short-term** smoking cessation rates relative to controls in non-clinical populations.[27] Our own work has demonstrated the short-term efficacy of a low-cost CM intervention among homeless shelter residents[2] and socioeconomically disadvantaged smokers participating in a safety net hospital smoking cessation program.[1] However, only a few studies have demonstrated superior smoking cessation outcomes for a CM intervention relative to controls at 6 month follow-up and beyond; these interventions focused on employees of a multinational company,[22] pregnant/post-partum women,[15,17] and smokers treated at a university clinic in Spain.[25] Notably, several other studies report no long-term cessation advantage for CM interventions.[20,26,28,29] Thus, it remains unclear whether CM interventions have a sustained impact on smoking cessation in general, and within socioeconomically disadvantaged populations specifically.

Although incentives for the prevention for chronic disease are now available through Medicaid and the Affordable Care Act, this approach has not been widely adopted for a variety of reasons including cost, complexity, and limited knowledge of how to apply and evaluate CM interventions in practice.[30-32] Clinics in medical settings are an ideal place to both target socioeconomically disadvantaged smokers and adapt the CM approach for clinical practice. Thus, the contribution of this trial will be to evaluate the longer-term (i.e., 6 months post-quit) effectiveness of offering low-cost financial incentives for biochemically verified abstinence among economically disadvantaged individuals who attend the Tobacco Treatment Research Program (TTRP; IRB# 6951) offered through the TSET Health Promotion Research Center; and to gain a greater understanding of CM treatment mechanisms and other contextual factors that influence abstinence in this vulnerable and understudied population. *This contribution is significant because it brings us closer to the development of practical, inexpensive, and effective smoking cessation interventions for socioeconomically disadvantaged smokers that may be applied in real-world settings; and it is a step towards understanding the factors that contribute to low cessation rates among socioeconomically disadvantaged smokers.* As CM interventions are incorporated into medical settings and relevant treatment targets are identified, we expect improved cessation interventions and increased smoking cessation rates among socioeconomically disadvantaged individuals. This could result in reductions in tobacco-related disease and health disparities, along with decreases in nationwide tobacco-attributable Medicaid expenditures which have been estimated to be $22 billion annually.[33]

C. Preliminary Studies/Progress Report

The PREVAIL pilot study was designed to evaluate the feasibility and **short-term** effectiveness (4 and 12 weeks post-quit) of offering small financial incentives (gift cards) for biochemically verified abstinence as an

IRB NUMBER: 6260
IRB APPROVAL DATE: 04/08/2021
IRB NUMBER: 6260
IRB APPROVAL DATE: 04/08/2021

adjunct to the smoking cessation program offered at Parkland Hospital (Dallas County safety net hospital). **A manuscript describing PREVAIL pilot study outcomes was recently published in the *American Journal of Public Health*.**[1] The standard tobacco treatment program at Parkland Hospital included an initial education/orientation session, weekly support groups facilitated by social workers, and appointments with a physician as needed to receive pharmacological treatment and individual follow-up. Interested and eligible individuals newly enrolled in the Parkland smoking cessation program (*N* = 146) were randomized to either Usual Care (UC; *n* = 71) or 4 weeks of CM (UC + adjunctive financial incentives; *n* = 75). All participants were followed weekly from 1 week pre-quit through 4 weeks post-quit; and a sub-sample (*N* = 128) was additionally asked to complete an in-person follow-up visit at 12 weeks post-quit. Participants were assessed weekly for smoking status from 1 week pre-quit through 4 weeks post-quit when they attended treatment visits, and they earned $30 for completing each of 4 assessments (i.e., baseline, quit date, 1 week post-quit, and 4 weeks post-quit) completed on a laptop computer. Individuals randomized to the CM intervention additionally had the opportunity to earn gift cards for self-reported and biochemically-verified abstinence at each visit, such that participants could earn $20 in gift cards for quitting on the specified quit day (i.e., one week after the orientation visit), and this amount increased by $5 with each successive abstinent visit (i.e., up to $40 in gift cards at 4 weeks post-quit; $150 total). **Note that we have also used this identical incentives schedule successfully with a small sample of homeless smokers in a shelter setting.**[2] Participants in the CM group were significantly more likely to achieve 7-day point prevalence abstinence than UC at all visits after the quit date (all *p*'s < .05). At 4 weeks post-quit, 49.3% of CM participants were abstinent vs. 25.4% of those randomized to UC, *p*=.001; OR=3.40; 95% CI, 1.61, 7.16). In the subsample (*N* = 128) that had the opportunity to complete the follow-up visit at 12 weeks post-quit, 32.8% achieved point prevalence abstinence in the CM group vs. 14.1% of those randomized to UC (*p*<.01, OR=3.76, 95% CI, 1.45, 9.70).

D. Research Design and Methods

Description of Standard Care. According to the Clinical Practice Guideline,[34] currently recommended components of an intensive tobacco treatment intervention include 1) initial assessment of willingness to participate, 2) the use of multiple types of clinicians (e.g., medical, non-medical), 3) at least 4 treatment sessions, in an individual counseling format, that are greater than 10 minutes in duration, 4) counseling that includes problem-solving, skills training, and social support components, and 5) and the opportunity to use effective medications to aid in tobacco cessation (e.g., nicotine patch, varenicline). The TTRP currently offers all components of an intensive tobacco treatment intervention. Specifically, individuals who would like to quit smoking are referred to the TTRP (via electronic medical record, telephone call, or email). Patients are scheduled for their initial appointment for an individual counseling session and to discuss pharmacological treatment options. Nicotine replacement therapy is provided during the session, or other medications are prescribed by a prescribing provider through the TTRP. Additional individual counseling sessions are offered once per week. Those enrolled in the current study will be encouraged to attend at least 5 weekly group sessions to facilitate follow-up and the delivery of study incentives.

Recruitment/Screening (Visit 1, Part 1; Screening). Individuals who are referred to the TTRP for smoking cessation treatment and report that they are uninsured or receiving Medicaid benefits and can speak, read, and understand English will be screened. In addition, Trialfacts will assist with recruitment by designing and managing targeted, GCP-compliant, IRB approved advertising. Potential participants who express an interest will click on a Trialfacts-generated ad and will be taken to a custom landing page created by Trialfacts, where they will be screened for eligibility (called pre-screening) and those who pass the Trialfacts online pre-screening questionnaire may be referred to the study team. Participants who report that they cannot read, speak, and understand English during the recruitment or screening sections will be excluded as participants must be able to answer assessments on their own using the smartphone. Additionally, participants who are not U.S. Citizens or Permanent Residents (who must supply a copy of their green card as proof of residency) or individuals who are unwilling/unable to provide their residency status, SSN, and whether they are a University of Oklahoma employee will be excluded because they cannot be paid using gift cards according to the University of Oklahoma Administrative Policy Part 500 Section 557 and this study intervention relies on immediate compensation. At the first visit, study staff will inquire about their interest in finding out more about the study. Study staff will review the consent form with interested participants, and they will be screened for

IRB NUMBER: 6260
IRB APPROVAL DATE: 04/08/2021
IRB NUMBER: 6260
IRB APPROVAL DATE: 04/08/2021

eligibility on-site in a private room in the clinic. Participant eligibility for the current study will not influence eligibility for the TTRP. The *Rapid Estimate of Adult Literacy in Medicine* (REALM; see Appendix A) will be administered to ensure that all participants are able to read at ≥ sixth grade level (i.e., required to complete EMA and self-report questionnaires). Expired carbon monoxide (CO) will be measured with a Vitalograph BreathCO carbon monoxide monitor. Participants will be questioned about their 1) insurance status 2) age, 3) current level of smoking, 4) willingness to quit smoking, 5) willingness/ability to attend 6 weekly sessions (including the first visit), and 6) their residency status, SSN and whether they are University of Oklahoma employees. Ineligible participants who enroll in the Tobacco Treatment Research Program (IRB 6951) will receive a $50 gift card. Eligible participants may complete the assessment portion of visit 1.

Pre-Quit (Visit 1, Part 2; Assessment). Participants will complete self-report questionnaires on a laptop computer; and expired CO, weight, and height will be measured in a private room to ensure confidentiality. Participants will be provided with an Android smartphone and instructed regarding the use of the phone (participants may make personal calls) as well as the EMA procedures. Participants will receive 4 random prompts and 1 daily diary prompt (in the morning) during the normal waking hours each day for four consecutive weeks. Participants will be instructed to quit smoking at bedtime or 10:00 pm (whichever occurs first), on the evening prior to their next scheduled TTRP session. Participants will be randomized to Standard Care (SC) or CM using a random numbers table and advised of their group assignment at the conclusion of visit 1. The appropriate schedule of payments (SC or CM) will be discussed with all participants (see Table 1). Participants will receive a $50 payment for completion of the in-person assessments at visit 1 which takes approximately 1 hour to complete, and participants will be scheduled to return for visit 2.

Quit Day (Visit 2). Participants will complete self-report questionnaires on a laptop computer; and expired CO and weight will be measured in a private room to ensure confidentiality. All participants will be paid $30 for the completion of the in-person assessments at visit 2, which take approximately 40 minutes to complete. Participants randomized to the CM treatment will receive an additional $20 payment if they self-report abstinence from smoking since 10 p.m. the prior evening, and their expired is CO level is < 10 ppm (please note that the less stringent cut-off of 10 ppm will be used to verify abstinence at the first visit only, due to the recency of quitting). Participants who do not attend will be contacted by phone to obtain their self-reported smoking status.

1 Week Post-Quit (Visit 3). Participants will complete self-report questionnaires on a laptop computer; and expired carbon monoxide (CO) and weight will be measured in a private room to ensure confidentiality. In addition, participants will receive $30 for the completion of the in-person assessment at visit 3, which takes approximately 30 minutes to complete. Participants randomized to the CM treatment will receive an additional $25 payment if they self-report continuous abstinence from smoking since the quit day and have an expired CO level of ≤ 6 ppm. **2 Weeks Post-Quit (Visit 4).** Expired carbon monoxide (CO) and weight will be measured in a private room to ensure confidentiality. Participants will receive $30 in gift cards for the completion of the assessment, which will take approximately 5 minutes to complete. Participants randomized to the CM treatment will receive $30 payment if they self-report continuous abstinence from smoking since the quit date and have an expired CO level of ≤ 6 ppm. **3 Weeks Post-Quit (Visit 5).** Expired carbon monoxide (CO) and weight will be measured in a private room to ensure confidentiality. Participants will receive $30 in gift cards for the completion of the assessment, which will take approximately 5 minutes to complete. Participants randomized to the CM treatment will receive $35 payment if they self-report continuous abstinence from smoking since the quit date and have an expired CO level of ≤ 6 ppm. **4 Weeks Post-Quit (Visit 6).** Participants will complete self-report questionnaires on a laptop computer; and expired CO and weight will be measured in a private room to ensure confidentiality. Participants will receive a $30 payment for completion of the in-person assessments, which will take approximately 50 minutes to complete. Upon return of the smartphones, participants will be compensated based on the percentage of completed random assessments. Specifically, those who complete 50%-74% of assessments will receive $75 in gift cards, those who complete 75%-89% of assessments will receive $100 in gift cards, and those who complete 90% or more of their assessments will receive $150 in gift cards. Participants who do not attend will be contacted by phone and/or mail to request that the phone be returned by mail (postage paid envelopes will be sent) or in-person at the next visit. When participants return the phone they will be compensated according to the compensation schedule described above. Participants whose only form of telephone contact is the study phone will be

IRB APPROVED
IRB NUMBER: 6260
IRB APPROVAL DATE: 04/08/2021
IRB APPROVED
IRB NUMBER: 6260
IRB APPROVAL DATE: 04/08/2021

allowed to keep and use the study phone for their remaining duration of the study (up to their 26-week post-quit visit). These participants will still be compensated for EMA completion on the same schedule as any other participant and they will be asked to complete the same number of EMA assessments as any other participant. Participants randomized to the CM treatment will receive an additional $40 payment if they self-report continuous abstinence from smoking since the quit date and have an expired CO level of ≤ 6 ppm. Participants who do not attend will be contacted by phone to obtain their self-reported smoking status.

8 Weeks Post-Quit (Visit 7). Participants will complete self-report questionnaires on a laptop computer; and expired CO and weight will be measured in a private room to ensure confidentiality. Participants will receive a $40 payment for completion of the in-person assessments, which will take approximately 30 minutes to complete. Participants randomized to the CM treatment will receive an additional $50 payment if they self-report abstinence from smoking over the past 7 days and have an expired CO level of ≤ 6 ppm. Participants who do not attend will be contacted by phone to obtain their self-reported smoking status.

12 Weeks Post-Quit (Visit 8). Participants will complete self-report questionnaires on a laptop computer; and expired CO and weight will be measured in a private room to ensure confidentiality. Participants will receive a $40 payment for completion of the in-person assessments, which will take approximately 30 minutes to complete. Participants randomized to the CM treatment will receive an additional $50 payment if they self-report abstinence from smoking over the past 7 days and have an expired CO level of ≤ 6 ppm. Participants who do not attend will be contacted by phone to obtain their self-reported smoking status.

26 Weeks Post-Quit (Visit 9; Follow-Up). Participants will complete self-report questionnaires on a laptop computer; and expired CO and weight will be measured in a private room to ensure confidentiality. Participants will receive a $40 payment for completion of the in-person assessments, which will take approximately 40 minutes to complete. Saliva cotinine will also be measured via NicAlert test strips to provide additional evidence of abstinence. Participants who do not attend will be contacted by phone to obtain their self-reported smoking status and mailed a saliva cotinine test with instructions on how to complete the test.

Remote Treatment Follow-up Visits. Due to the evolving circumstances regarding COVID-19, participants will complete each follow-up visit remotely. The requirements and compensation for remote visits will be identical to in-person visits. Participants will remotely complete web-based questionnaires and provide a smartphone-based CO measurement using a Smokerlyzer iCO monitor. Participants randomized to the CM treatment will receive incentives in accordance with each visit given they meet the cessation requirements outlined above.

MEASURES

Biological/Anthropometric Measures.

Expired Carbon Monoxide (CO) levels will be measured with a portable Vitalograph ecolyzer. CO levels of ≥ 8-10 parts per million (ppm) suggest recent cigarette smoking with a sensitivity and specificity of approximately 90 percent. Thus, individuals will be eligible to participate in the current study if they produce CO levels ≥ 8 ppm at the screening visit. Conversely, abstinence from smoking will be verified with a CO level of < 10 on the quit day, and ≤ 6 ppm at all subsequent post-quit visits.

Saliva Cotinine will be measured with Accutest NicAlert test strips at the final follow-up visit (26 weeks post-quit). Cotinine levels of <10 ng/ml suggest abstinence from smoking, while levels of 10 ng/ml or greater are consistent with smoking. Cotinine levels will be recorded by research staff as it becomes available on the test strip. After the result is recorded, test strips and saliva will be immediately discarded in a biohazard container.

Body Mass Index (BMI) will be calculated (kg/m²) based on height measurements collected at visit 1 and weight measurements collected at each visit.

Traditional Questionnaire Measures.

Questionnaire data will be collected primarily on laptop computers. The amount of time needed to complete the computer-administered questionnaires varies by study visit. It is estimated that the longest visit (baseline) will require 1 hour to complete. We have found that even individuals with no experience

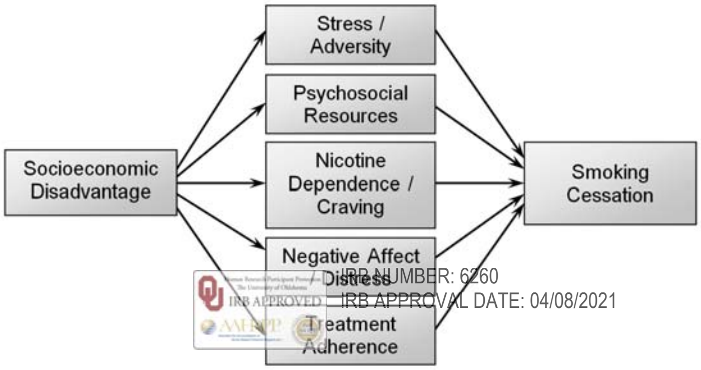

Figure 1. **Conceptual model of the influence of socioeconomic disadvantage on smoking cessation.**

IRB NUMBER: 6260
IRB APPROVAL DATE: 04/08/2021
8/2021

using computers have had few problems completing study assessments. We will include measures that are consistent with our working conceptual model (Figure 1). See Appendix A for assessment schedule and questionnaire measures.

Ecological Momentary Assessment. Participants will be trained in the EMA procedures at the baseline (1 week pre-quit) visit. Assessment items were selected based on their hypothesized relations to smoking behavior, temptation and lapse episodes, and/or their potential importance in understanding the influence of socioeconomic disadvantage. The items will assess smoking urges, affect, expectancies, self-efficacy, and related constructs. EMA items are detailed in the Appendix B.

Hardware. The Samsung Galaxy Exhibit smart phone uses the Android 4.1 Jellybean operating system. The phone has a 3.8 inch (480x800 pixel resolution) touch screen display, a built in microphone, earphone jack, audio speaker, and a rechargeable battery with 9.0 hours of talk time. It is Wi-Fi and GPS capable. Participants navigate through the EMA program and enter data simply by touching the screen, similar to a pen-and-paper questionnaire. Thus, computer/typing skills are not required. Participants have the ability to call (e.g., if they have problems completing EMAs) and receive calls from research staff through the smart phone free of charge. The smart phones are equipped with a Secure Digital card to store data as it is collected.

Programming. The EMA program will be developed by and accessed through the mHealth core led by Michael Businelle (co-investigator). The core is located in the TSET Health Promotion Research Center and is part of the Stephenson Cancer Center. The EMA program that was developed for the PREVAIL pilot study will be modified for the purposes of the proposed study.

EMA Assessment Types. Three types of assessments will be used: daily diary, random sampling, and event sampling (i.e., pre-cessation smoking, urge, post-cessation lapse). Daily diary assessments will be completed once every day (30 minutes after waking), and all questions will refer to the previous 24 hours. Random sampling and daily diary assessments will be initiated by the smart phone. The phone will audibly and visually cue each random assessment for 30 seconds. If the participant has not responded after three prompts, the assessment will be recorded as missed. Random sampling will include four smart phone initiated assessments per day, scheduled to occur during each participant's normal waking hours. Event sampling is initiated by participants. Participants are instructed to complete "smoking assessments" when they smoke prior to the quit date, "urge assessments" when they have an urge to smoke or they feel like they almost smoked, and "lapse assessments" if they smoke after the quit date. GPS coordinates (i.e., latitude and longitude) will be collected via the smartphone during each assessment. On average, random and event sampling assessments will take 2.5 minutes to complete, while daily diary assessments will take approximately 5 minutes to complete. All assessments will be date and time stamped for future analyses.

Daily Diary. Daily Diary Assessments will be completed daily from 1 week prior to the quit date through 3 weeks post-quit (4 weeks total) and all questions will query about the previous day. Smoking rate and alcohol consumption will be assessed with the items "How many cigarettes did you smoke yesterday?" and "Did you drink any alcohol yesterday?" If the participant answers "yes" to the alcohol item, he/she will be prompted to indicate the type and quantity of the alcohol that was consumed." The Perceived Stress Scale will also be included in the daily diary assessment and it is identical to the questionnaire measure, with the exception that each item will be rated based on the past 24 hours rather than the past 7 days. Items that assess social support, discrimination, discrete stressors, risk perception, and reasons for quitting will also be included in the daily diary assessments.

Random Sampling. Participants will be prompted to complete random assessments 4 times each day from 1 week pre-quit date through 3 weeks post-quit (i.e., 4 weeks total). Participants will rate their affect by indicating the extent to which they agree or disagree with each of eleven statements: I feel irritable, happy, content, frustrated/angry, sad, worried, miserable, restless, stressed, hostile, and calm (based on the circumplex model of affect). In addition, participants will be asked to describe their current environment (e.g., at home, work, outside) and if they are alone or with others. Smoking urges, cigarette availability, smoking expectancies, motivation for abstinence, and abstinence self-efficacy will also be assessed during random sampling.

Event Sampling. Smoking Assessments. During the first week of assessment (i.e., the pre-quit

IRB NUMBER: 6260
IRB APPROVAL DATE: 04/08/2021
IRB NUMBER: 6260
IRB APPROVAL DATE: 04/08/2021

week), participants will be instructed to click the “record cigarette” button immediately prior to smoking each cigarette. Because the assessment burden would be excessive for heavy smokers if each smoking occasion were assessed, two of these smoking occasions each day will be randomly sampled for pre- and post-cigarette assessments. Ten minutes after completing the pre-cigarette assessment, the smartphone will automatically prompt the post-cigarette assessment which includes unique items and the core EMA items. Urge Assessments. Following their quit date, participants will be instructed to initiate an urge assessment each time they “experience an urge to smoke” and answers should be focused on their immediate thoughts/feelings. Lapse Assessments. Participants will be instructed to complete lapse assessments each time they smoke after the quit date. Questions asked during lapse assessments are nearly identical to those presented in random and urge assessments. However, questions are worded to separately assess the participant’s responses immediately prior to AND following the lapse. Post lapse assessments also query about recent discrimination, the reinforcing value of the lapse cigarette(s), and lapse causes.

E. Statistical Methods

Aim 1. The sample size calculation (*N* = 320) assumes a 30% drop-out rate for both treatment arms. The number of subjects who will complete the 26-week trial (*n* = 112 in each group) was estimated based on the following assumptions: 1) SC abstinence rate is 10% at 26-weeks post-quit, 2) CM abstinence rate is 22% at 26 weeks post-quit; 3) equal allocation of subjects between two treatments; 4) type I error rate is set as 0.05; and 5) the targeted minimum power is 0.8. Logistic regression analyses will be conducted to evaluate the influence of adjunctive CM relative to SC on long-term smoking cessation (binary variable) outcomes at 26 weeks post-quit. The impact of treatment group on smoking cessation at secondary endpoints including the quit day, and at 1, 2, 3, 4, 8, and 12 weeks will also be evaluated. Generalized linear mixed models may also be used to evaluate the influence of treatment group on smoking cessation as a repeated outcome measure (see analysis plan in Aim 2). Covariates may include age, gender, race/ethnicity, nicotine dependence, and pharmacological treatment. As is the standard in smoking cessation intervention research, a conservative intention-to-treat approach will be employed in the primary analyses, such that participants with missing data or who have dropped out of treatment will be considered non-abstinent. Less conservative “completer” analyses will also be conducted where only participants with complete smoking status data will be included (i.e., those with missing data will be excluded).

Aim 2. The detectable effects for analyses based on a complete sample size of 320, 2-sided tests, alpha=.05, and power=.80 are presented in Table 3. Where appropriate, these estimates account for the fact that repeated observations from the same person will be correlated. The most conservative detectable effects for the GLMM EMA analyses will be presented. That is, the case with a single type of assessment (daily diary assessments) that occurs with the least frequency (once a day). Thus, calculations are based on an N of 320 completing 80% of 28 assessments during the study period (7 assessments/week X 4 weeks) for a total of 7168 assessments across all participants. Detectable effects for the other types of EMA assessments were not computed because all else remaining equal, detectable effects for the variables collected during these assessment types (e.g., random assessments) will be smaller because of the larger number of assessments. A range of intraclass correlation coefficient (ICC) values were chosen based on our previous research, which yielded ICC estimates of .35 and .44 for mood and urge ratings during random assessments. Detectable effects estimates depend on the variance inflation factor (VIF) which is based on both the size of the ICC and the average number of observations per person. The VIF is used to calculate an effective sample size (Table 2). Detectable group differences were based on varying group distributions and are presented for both dichotomous and continuous variables at varying ICC values. Given our sample size, we can detect a factor responsible for improving the proportion who successfully abstained by as little as 2 percentage points. The average detectable improvement is 7 percentage points. Since relevant covariates will be adjusted for in all analyses, the actual detectable effects will be smaller than calculated. Logistic regression analyses of traditional and EMA data will be conducted to identify significant demographic, psychosocial, environmental, and behavioral predictors of short and longer-term smoking cessation (binary outcomes). Covariates may

Table 2. Detectable Effects for EMA Data.

IRB NUMBER: 6260
IRB APPROVAL DATE: 04/08/2021
IRB NUMBER: 6260
IRB APPROVAL DATE: 04/08/2021

ICC	VIF	Effective sample size	Detectable mean difference effects based on varying distributions: Dichotomous predictors .50; .50	.30; .70	.10; .90	Continuous Predictors Standard Normal Dbn
0.1	3.14	2282	0.042	0.046	0.070	0.021
0.3	7.42	1993	0.067	0.072	0.111	0.033
0.7	15.98	949	0.102	0.109	0.168	0.051

Note: All detectable effects are presented in standard deviation units and are at 80% power.

include treatment group, age, gender, race/ethnicity, nicotine dependence, and pharmacological treatment.
Generalized Linear Mixed Models. Smoking cessation assessment data and EMA data will be measured repeatedly and are therefore correlated within subjects. Thus, our analytic approach will include generalized linear mixed model regression analysis (GLMM). GLMM is a flexible analytic approach with wide use in health sciences research. GLMM can handle fixed and random effect model parameters, nested designs, and repeated measures with various correlation structures. GLMM can also handle normal and non-normal outcomes such as dichotomous smoking status variables, different variance functions, as well as unbalanced designs where the number of repeated observations varies across individuals. EMAs create an enormous amount of data. For instance, in the 2 weeks that PREVAIL participants carried the EMA device, we collected over 70 assessments per participant. This rich data will allow us to address multiple within and between subjects questions in this understudied and vulnerable group of low SES smokers. For example, we will examine key EMA variables (e.g., negative affect, smoking urge, stress) and variable parameters (e.g., intercept, slope, quadratic term, volatility) as potential predictors of week 26 smoking abstinence. In addition, we will identify key EMA variable parameters that predict specific lapse episodes (e.g., the first lapse following the quit date). This information may be used to detect high risk for relapse situations that may be targeted in future "smart interventions." EMA data will also allow us to address other important participant specific (i.e., within subjects) questions like: 1) what psychosocial changes occur as an individual progresses from lapse to relapse, and 2) what impact specific episodic events like exposure to stressors (e.g., discrimination events, acute urges) have on cessation self-efficacy and mood and the impact on smoking lapse.
Mediation. The PROCESS macro for SPSS/SAS will be used to conduct mediation analyses with the goal of identifying variables that mediate the relation between treatment condition and smoking cessation outcomes. This method uses an ordinary least squares path analytic framework to estimate direct and indirect effects in single and multiple mediation models, and bootstrapping methods are incorporated to generate confidence intervals. The macro can also be used to evaluate complex moderated mediation models including those with dichotomous outcomes (e.g., abstinence vs. non-abstinence), along with numerous other model variations.

F. Gender/Minority/Pediatric Inclusion for Research

The study has no inclusion/exclusion criteria based on gender or race/ethnicity. However, please note that counseling in the TTRP is presently offered in English only. The TTRP is available to patients who are ≥ 18 years of age. Children < 18 years of age will be excluded from the study.

G. Human Participants

Subject Population. Participants will be up to 350 individuals (320 required) recruited during their first visit to the OUHSC Tobacco Treatment Research Program (TTRP). Interested participants may be included in the study if they: 1) are currently uninsured or receiving Medicaid benefits, 2) earn a score ≥ 4 on the REALM indicating > 6th grade English literacy level, 3) are willing to quit smoking 7 days from their first visit, 4) are ≥ 18 years of age, 5) have an expired CO level ≥ 8 ppm suggestive of current smoking, 6) are currently smoking ≥ 5 cigarettes per day, 7) are willing and able to attend 9 study visits and 8) are willing and able to provide their residency status (must be a U.S. Citizen or Permanent Resident with a valid green card), SSN and whether they are University of Oklahoma employees.

IRB APPROVED
IRB NUMBER: 6260
IRB APPROVAL DATE: 04/08/2021
IRB APPROVED
IRB NUMBER: 6260
IRB APPROVAL DATE: 04/08/2021

1. **Sources of Research Material.** Demographic, psychosocial, environmental and behavioral data will be collected via 1) traditional self-report questionnaires completed in-person on a laptop computer, and 2) EMA completed daily via smartphone (4 random assessments daily, 1 daily dairy, pre-quit smoking assessments, and post-quit urge and lapse assessments). GPS coordinates (i.e., latitude and longitude) will be captured at the time of each EMA assessment. Smoking status will be evaluated at each visit via expired carbon monoxide and in-person interview. Saliva cotinine will be measured via test strips at the final follow-up visit, values will be recorded, and strips will be discarded in a biohazard container. Measurement of height will be collected at the first visit and weight will be collected at each in-person assessment. Attendance will be noted by the research staff each week.
2. **Recruitment and Informed Consent.** Individuals will be referred from TTRP and recruited using Trialfacts. During the first visit, individuals will be provided with detailed information about the study and given the opportunity to have their questions answered within a private room to ensure confidentiality. Written informed consent will be obtained from those who are interested in participating. A certificate of confidentiality will be obtained.
3. **Risks.** Participation in this study poses minimal risk to participants. However, one potential, although unlikely, risk to participants is loss of confidentiality. The severity of harm in the case of loss of confidentially may range from mild to severe depending upon the individual and the specific circumstances. However, the risks of participation in the study are similar to that of participation in standard care, as loss of confidentially may be experienced in either case. The investigators are unaware of any risks associated with the use of the Vitalograph BreathCO monitor for the measurement of expired carbon monoxide levels.
4. **Protections against Risk.**
   General Procedures. Each participant will be assigned an identification number that will be utilized in place of names in all electronic and print data files. The file containing the links between participant names and identifiers will be kept in a separate password-protected file, which will be destroyed 12 months after the completion of the study. All print information, including informed consent, questionnaires will be stored in locked filing cabinets at the TSET Health Promotion Research Center. Electronic data (with names omitted) will be maintained on the investigators' computers, and all computers and electronic files will be password protected. Participants will complete smartphone assessments through an encrypted mobile application, and all data is automatically saved and sent to institutional servers in real-time. Anthropometric measures, CO, and saliva cotinine will be collected in a private room. All project staff will receive training focused on each of the following topics: 1) project rationale and objectives, 2) the informed consent process, 3) general data collection procedures (e.g., computer data collection, privacy), 4) measurement of anthropometric data, 5) use of the carbon monoxide ecolyzer, and 6) saliva cotinine measurement via test strips. A certificate of confidentiality will be obtained.
   EMA Confidentiality Procedures. The following features are designed to address smartphone/EMA/GPS data security issues: 1) the data stored on the smartphone device is in a SQLite database in a sandbox environment where read/write operations are only available through the programming application. No file or output is readable to end users, 2) a password (only known to researchers) is required to authenticate the current user before data can be downloaded from the smartphone device to the server, 3) the web browser application linking the principal investigator's computer to the database is on HTTPS protocol (SSL certificate with encryption) which will guarantee the data transfer from web browser to the backend database is well protected, and 5) the backend database is hosted by the University of Oklahoma Data Center in a secure setup.
   Triafacts. Trialfacts adheres to data security and privacy standards consistent with HIPAA. Referral information from Trialfacts may be imported to REDCap through an API as a new study record. Trialfacts will not have access to any OUHSC study related data. The Trialfacts data security plan has been reviewed and approved by OUHSC IT.
5. **Potential Benefits.** Potential benefits to participants include the possibility that the adjunctive CM intervention will have a beneficial impact on smoking cessation outcomes. In addition, the knowledge gained from this study may be utilized to improve our understanding of the barriers to quitting and predictors of relapse among socioeconomically disadvantaged individuals. Such information may facilitate

IRB NUMBER: 6260
IRB APPROVAL DATE: 04/08/2021
IRB NUMBER: 6260
IRB APPROVAL DATE: 04/08/2021

the development of more effective smoking cessation interventions that may be utilized within safety net hospitals and clinics.

6. **Risks in relation to Benefits.** The current study involves very minimal risk to participants, and the risks of study participation are similar to that of participation in standard care (e.g., loss of confidentiality). All participants will be already seeking standard care before they are provided with any information about the research study. Participants randomized to the financial incentives intervention may benefit from increased odds of smoking cessation, and all participants will be compensated for their time and effort. In addition, the knowledge gained from this study may be utilized to improve our understanding of the barriers to quitting and predictors of relapse among socioeconomically disadvantaged individuals.
7. ***Protections Against Risk.*** Participants will use a study smartphone or their personal smartphone to complete assessments through an encrypted mobile application and all data will be automatically saved and sent to the study server. For those who receive study smartphones, the research staff will use a unique Google Play Store login to download the study app onto the phone. Passwords will only be known to research staff. At the conclusion of the participant's time in the study, participants will return the phone and all data collection through the Insight application will end. Study data will then be removed from the study phone. Participants who use their personal mobile device will use their personal Google Play Store account to download the Insight app. At the conclusion of the participant's time in the study, the study data will be removed from the participant's phone and all data collection through the Insight application will end. Researchers will also give participants instructions on how to delete the app from their personal device once they complete the study.

H. Data and Safety Monitoring Plan

The study poses minimal risk to participants, therefore continuous monitoring and reporting of events will be undertaken by the principal investigator (Dr. Kendzor) and co-investigators (Drs. Businelle, Swartz, and Wetter). Unanticipated problems will be promptly reported to the IRB. Since the standard smoking cessation treatment is offered by the Stephenson Cancer Center independent of this research proposal, adverse event monitoring will focus on events related to study assessments and the administration of financial incentives. Possible (though unlikely) adverse events might include compromised data security, and severe emotional reactions by participants due to non-payment of incentives following conflicting self-reports of abstinence and biochemical verification suggesting non-abstinence. Procedures to minimize the risk of loss of confidentiality are described in section G under the heading *Protections against Risk*.

IRB APPROVED
IRB NUMBER: 6260
IRB APPROVAL DATE: 04/08/2021
IRB APPROVED
IRB NUMBER: 6260
IRB APPROVAL DATE: 04/08/2021

I. Literature Cited

1. Kendzor DE, Businelle MS, Poonawalla IB, et al. Financial Incentives for Abstinence Among Socioeconomically Disadvantaged Individuals in Smoking Cessation Treatment. *Am J Public Health.* 2015/06/01 2015;105(6):1198-1205.
2. Businelle MS, Kendzor DE, Kesh A, et al. Small financial incentives increase smoking cessation in homeless smokers: A pilot study. *Addict Behav.* 3// 2014;39(3):717-720.
3. Jamal A, Agaku IT, O'Connor E, King BA, Kenemer JB, Neff L. Current cigarette smoking among adults — United States, 2005–2013. *MMWR Morb Mortal Wkly Rep.* 2014;63(47):1108-1112.
4. Kotz D, West R. Explaining the social gradient in smoking cessation: It's not in the trying, but in the succeeding. *Tob Control.* 2009;18(1):43-46.
5. Hiscock R, Bauld L, Amos A, Fidler JA, Munafò M. Socioeconomic status and smoking: a review. *Ann N Y Acad Sci.* 2012;1248(1):107-123.
6. Matthews KA, Gallo LC, Taylor SE. Are psychosocial factors mediators of socioeconomic status and health connections? *Ann N Y Acad Sci.* 2010;1186:146-173.
7. Matthews KA, Gallo LC. Psychological perspectives on pathways linking socioeconomic status and physical health. *Annu Rev Psychol.* 2011;62:501-530.
8. Dutra L, Stathopoulou G, Basden SL, Leyro TM, Powers MB, Otto MW. A meta-analytic review of psychosocial interventions for substance use disorders. *Am J Psychiatry.* 2008;165(2):179-187.
9. Prendergast M, Podus D, Finney J, Greenwell L, Roll J. Contingency management for treatment of substance use disorders: a meta-analysis. *Addiction.* 2006;101(11):1546-1560.
10. Stitzer M, Petry N. Contingency management for treatment of substance abuse. *Annual Review of Clinical Psychology.* 2006;2:411-434.
11. Dunn KE, Sigmon SC, Reimann EF, Badger GJ, Heil SH, Higgins ST. A Contingency-Management Intervention to Promote Initial Smoking Cessation Among Opioid-Maintained Patients. *Exp Clin Psychopharmacol.* 2010;18(1):37-50.
12. Dunn KE, Sigmon SC, Thomas CS, Heil SH, Higgins ST. Voucher based contingent reinforcement of smoking abstinence among methadone maintained patients: A pilot study. *J Appl Behav Anal.* 2008;41(4):527-538.
13. Alessi SM, Petry NM, Urso J. Contingency management promotes smoking reductions in residential substance abuse patients. *J Appl Behav Anal.* 2008;41(4):617-622.
14. Hunt YM, Rash CJ, Burke RS, Parker JD. Smoking cessation in recovery: Comparing 2 different cognitive behavioral treatments. *Addict Disord Their Treat.* 2010;9(2):64-74.
15. Heil SH, Higgins ST, Bernstein IM, et al. Effects of voucher-based incentives on abstinence from cigarette smoking and fetal growth among pregnant women. *Addiction.* 2008;103(6):1009-1018.
16. Higgins ST, Washio Y, Heil SH, et al. Financial incentives for smoking cessation among pregnant and newly postpartum women. *Prev. Med.* 2012;55, Supplement:S33-S40.
17. Higgins ST, Bernstein IM, Washio Y, et al. Effects of smoking cessation with voucher-based contingency management on birth outcomes. *Addiction.* 2010;105(11):2023-2030.
18. Donatelle RJ, Prows SL, Champeau D, Hudson D. Randomised controlled trial using social support and financial incentives for high risk pregnant smokers: Significant Other Supporter (SOS) program. *Tob Control.* September 1, 2000 2000;9(suppl 3):iii67-iii69.
19. Correia CJ, Benson TA. The use of contingency management to reduce cigarette smoking among college students. *Exp Clin Psychopharmacol.* 2006;14(2):171-179.
20. Tevyaw TOL, Colby SM, Tidey JW, et al. Contingency management and motivational enhancement: A randomized clinical trial for college student smokers. *Nicotine Tob Res.* 2009;11(6):739-749.
21. Volpp KG, Levy AG, Asch DA, et al. A randomized controlled trial of financial incentives for smoking cessation. *Cancer Epidemiol Biomarkers Prev.* 2006;15(1):12-18.
22. Volpp KG, Troxel AB, Pauly MV, et al. A randomized, controlled trial of financial incentives for smoking cessation. *N Engl J Med.* 2009;360(7):699-709.
23. Stoops WW, Dallery J, Fields NM, et al. An Internet-based abstinence reinforcement smoking cessation intervention in rural smokers. *Drug Alcohol Depend.* 2009;105(1-2):56-62.

IRB NUMBER: 6260
IRB APPROVAL DATE: 04/08/2021
IRB NUMBER: 6260
IRB APPROVAL DATE: 04/08/2021

24. Hertzberg JS, Carpenter VL, Kirby AC, et al. Mobile Contingency Management as an Adjunctive Smoking Cessation Treatment for Smokers With Posttraumatic Stress Disorder. *Nicotine Tob Res.* November 1, 2013 2013;15(11):1934-1938.
25. Secades-Villa R, García-Rodríguez O, López-Núñez C, Alonso-Pérez F, Fernández-Hermida JR. Contingency management for smoking cessation among treatment-seeking patients in a community setting. *Drug Alcohol Depend.* 7/1/ 2014;140(0):63-68.
26. Ledgerwood DM, Arfken CL, Petry NM, Alessi SM. Prize contingency management for smoking cessation: A randomized trial. *Drug Alcohol Depend.* 2014.
27. Giles EL, Robalino S, McColl E, Sniehotta FF, Adams J. The effectiveness of financial incentives for health behaviour change: Systematic review and meta-analysis. *PLoS ONE.* 2014;9(3).
28. Dallery J, Raiff BR, Grabinski MJ. Internet-based contingency management to promote smoking cessation: A randomized controlled study. *J Appl Behav Anal.* 2013;46(4):750-764.
29. Glasgow RE, Hollis JF, Ary DV, Boles SM. Results of a year-long incentives-based worksite smoking-cessation program. *Addict Behav.* 7// 1993;18(4):455-464.
30. Hartzler B, Lash SJ, Roll JM. Contingency management in substance abuse treatment: A structured review of the evidence for its transportability. *Drug Alcohol Depend.* 4/1/ 2012;122(1–2):1-10.
31. Blumenthal KJ, Saulsgiver KA, Norton L, et al. Medicaid Incentive Programs To Encourage Healthy Behavior Show Mixed Results To Date And Should Be Studied And Improved. *Health Aff.* March 1, 2013 2013;32(3):497-507.
32. Carroll KM. Lost in translation? Moving contingency management and cognitive behavioral therapy into clinical practice. *Ann N Y Acad Sci.* 2014:n/a-n/a.
33. Armour BS, Finkelstein EA, Fiebelkorn IC. State-level Medicaid expenditures attributable to smoking. *Prev Chronic Dis.* 2009;6(3).
34. Fiore MC, Jaen CR, Baker TB, Bailey WC, Benowitz NL, Curry SJ. Treating Tobacco Use and Dependence: 2008 Update. *Clinical Practice Guideline* 2008; http://www.ahrq.gov/professionals/clinicians-providers/guidelines-recommendations/tobacco/clinicians/treating_tobacco_use08.pdf.

IRB APPROVED
IRB NUMBER: 6260
IRB APPROVAL DATE: 04/08/2021
IRB APPROVED
IRB NUMBER: 6260
IRB APPROVAL DATE: 04/08/2021
