# Supplementary material for: Financial Incentives for Smoking Cessation Among Socioeconomically Disadvantaged Adults: A Randomized Clinical Trial
Source: JAMA Netw Open. 2024 Jul 2;7(7):e2418821. doi: 10.1001/jamanetworkopen.2024.18821 (PMC11220567; doi:10.1001/jamanetworkopen.2024.18821)
Supplement: Supplement 2. — eTable. Comparison of the Baseline Characteristics of Participants Who Completed the Final 26-Week Follow-Up vs Those Who Did Not [file jamanetwopen-e2418821-s002.pdf]

Supplementary Online Content

Kendzor DE, Businelle M, Frank-Pearce SG, et al. Financial incentives for smoking cessation among socioeconomically disadvantaged adults: a randomized clinical trial. *JAMA Netw Open*. 2024;7(7):e2418821. doi:10.1001/jamanetworkopen.2024.18821

eTable. Comparison of the Baseline Characteristics of Participants Who Completed the Final 26-Week Follow-Up vs Those Who Did Not

This supplementary material has been provided by the authors to give readers additional information about their work.

© 2024 Kendzor DE et al. *JAMA Network Open*.

eTable. Comparison of the Baseline Characteristics of Participants Who Completed the Final 26-Week Follow-Up vs Those Who Did Not

	Completed (*n*=209)	Missing (*n*=111)	*p*
Age, Years, *M* (SD)[a]	50.2 (11.5)	46.5 (11.6)	**0.0072**
Sex, Female, *n* (%)	144 (68.9%)	58 (52.3%)	**0.0033**
Sexual/Gender Minoritized, *n* (%)[b]	25 (12.0%)	15 (13.5%)	0.6895
Race			0.2221
White, *n* (%)	133 (63.6%)	67 (60.4%)	
Black/African American, *n* (%)	55 (26.3%)	27 (24.3%)	
American Indian/Alaska Native, *n* (%)	5 (2.4%)	8 (7.2%)	
Multi-Race/Other, *n* (%)[c]	16 (7.7%)	9 (8.1%)	
Ethnicity, Hispanic, *n* (%)	8 (3.8%)	7 (6.3%)	0.3181
Racially/Ethnically Minoritized, *n* (%)	80 (38.3%)	46 (41.4%)	0.5814
Education, <High School, *n* (%)[a]	43 (20.6%)	19 (17.1%)	0.4446
Annual Household Income, <$11,000, *n* (%)[d]	117 (56.0%)	58 (52.3%)	0.5950
Health Insurance, Medicaid or Medicaid Combination, *n* (%)[a,e]	83 (39.7%)	61 (55.0%)	**0.0101**
Any Study Participation during COVID-19 Pandemic, n (%)[f]	83 (39.7%)	63 (56.8%)	**0.0036**
Cigarettes Smoked Per Day (pre-quit), *M* (SD)[a]	18.6 (9.7)	20.1 (10.7)	0.2262
Years of Smoking, *M* (SD)[a]	30.7 (12.9)	26.3 (13.1)	**0.0039**
Expired CO (pre-quit), ppm, *M* (SD)	22.3 (11.7)	22.8 (11.5)	0.7281
Heaviness of Smoking Index, score ≥5, *n* (%)[a]	41 (19.6%)	22 (19.8%)	0.9815

Note: Bolded values indicate statistically significant differences (p<0.05).
[a]1 participant did not provide this information.
[b]Participants were considered sexual/gender minoritized if they identified as lesbian or gay (*n*=12), bisexual (*n*=19), transgender (*n*=3), did not know or were not sure about their sexual orientation (*n*=2), or chose not to respond (*n*=5). Those who identified as straight and did not identify as transgender were considered heterosexual/cisgender.
[c]15 participants who identified as Multi-Race selected American Indian/Alaska Native as one of their races.
[d]9 participants did not provide this information.
[e]Participants who did not have Medicaid insurance were uninsured.
[f]Participants enrolled between September 2019 and August 2021 had at least 1 study visit scheduled during the COVID-19 pandemic.

© 2024 Kendzor DE et al. *JAMA Network Open*.
