# Supplementary material for: Financial Incentives for Smoking Cessation Among Socioeconomically Disadvantaged Adults: A Randomized Clinical Trial
Source: JAMA Netw Open. 2024 Jul 2;7(7):e2418821. doi: 10.1001/jamanetworkopen.2024.18821 (PMC11220567; doi:10.1001/jamanetworkopen.2024.18821)
Supplement: Supplement 3. — Data Sharing Statement [file jamanetwopen-e2418821-s003.pdf]

Data Sharing Statement

Kendzor. Financial Incentives for Smoking Cessation Among Socioeconomically Disadvantaged Adults. *JAMA Netw Open*. Published July 02, 2024.
doi:10.1001/jamanetworkopen.2024.18821

Data
Data available: Yes
Data types: Deidentified participant data, Data dictionary
How to access data: Data will be made available upon request by emailing darla-kendzor@ouhsc.edu.
When available: With publication

Supporting Documents
Document types: None

Additional Information
Who can access the data: Researchers and others with university approved requests.
Types of analyses: All reasonable requests will be entertained.
Mechanisms of data availability: We will follow all university requirements for data sharing with individuals who have reasonable data requests.
